# Infant trunk posture and arm movement assessment using pressure mattress, inertial and magnetic measurement units (IMUs)

**DOI:** 10.1186/1743-0003-11-133

**Published:** 2014-09-06

**Authors:** Andraž Rihar, Matjaž Mihelj, Jure Pašič, Janko Kolar, Marko Munih

**Affiliations:** Faculty of Electrical Engineering, University of Ljubljana, 25, Tržaška cesta, 1000 Ljubljana Slovenia

**Keywords:** Infant trunk posture, Arm movement assessment, Pressure mattress, Inertial magnetic measurement units

## Abstract

**Background:**

Existing motor pattern assessment methods, such as digital cameras and optoelectronic systems, suffer from object obstruction and require complex setups. To overcome these drawbacks, this paper presents a novel approach for biomechanical evaluation of newborn motor skills development. Multi-sensor measurement system comprising pressure mattress and IMUs fixed on trunk and arms is proposed and used as alternative to existing methods. Observed advantages seem appealing for the focused field and in general. Combined use of pressure distribution data and kinematic information is important also for posture assessment, ulcer prevention, and non-invasive sleep pattern analysis of adults.

**Methods:**

Arm kinematic parameters, such as root-mean-square acceleration, spectral arc length of hand velocity profile, including arm workspace surface area, and travelled hand path are obtained with the multi-sensor measurement system and compared to normative motion capture data for evaluation of adequacy. Two IMUs per arm, only one IMU on upper arm, and only one IMU on forearm sensor placement options are studied to assess influence of system configuration on method precision. Combination of pressure mattress and IMU fixed on the trunk is used to measure trunk position (obtained from mat), rotation (from IMUs) and associated movements on surface (from both). Measurement system is first validated on spontaneous arm and trunk movements of a dedicated baby doll having realistic anthropometric characteristics of newborns. Next, parameters of movements in a healthy infant are obtained with pressure mattress, along with trunk and forearm IMU sensors to verify appropriateness of method and parameters.

**Results:**

Evaluation results confirm that full sensor set, comprising pressure mattress and two IMUs per arm is a reliable substitution to optoelectronic systems. Motor pattern parameter errors are under 10% and kinematic estimation error is in range of 2 cm. Although, use of only forearm IMU is not providing best possible kinematic precision, the simplicity of use and still acceptable accuracy are convincing for frequent practical use. Measurements demonstrated system high mobility and usability.

**Conclusions:**

Study results confirm adequacy of the proposed multi-sensor measurement system, indicating its enviable potential for accurate infant trunk posture and arm movement assessment.

**Electronic supplementary material:**

The online version of this article (doi:10.1186/1743-0003-11-133) contains supplementary material, which is available to authorized users.

## Introduction

Early infancy is an important segment of infant’s life, as during the first six months infants gradually achieve some main developmental milestones and set the foundations for their upcoming life [1]. Reliable infant’s motor pattern assessment can ensure detection of atypical development [2] and subsequent early intervention, which in case of developmental disorders holds an important role in effective rehabilitation [3, 4].

Typically applied *clinical methods* for assessment of developmental patterns, such as AIMS, TIMP, and Bayley III [5], despite comprehensive knowledge, experience, and keen eye of clinicians, lack objectivity and precision. To avoid such drawbacks and ensure reliable, repeatable, and accurate results, use of measurement systems with optical, inertial, and other similar motion capture sensors, seems sensible and reasonable. Although, it has received notable attention in the last decades [6], widespread use is limited by specific anthropometric characteristics of infants, such as shorter segment lengths, lower weight, and intolerance to longer, invasive measurement sessions with complex setup preparation routines [7].

*Digital camera systems* have been used with additional videotape coding and classification, such as Observer coding program to study the influence of postural control on hand behaviour [8], and infant’s reaching behaviour in relation to hand preference [9]. Precision was improved with use of reflexive markers and integration of Dvideow image analysis system [10] for reconstruction of 3D head movement [11] and goal-directed reaching behaviour [12]. Digital camera systems are low-cost, but suffer from object obstruction, need of complex camera calibration, illumination of markers, and careful adjustments of zoom and focus [11].

*Optoelectronic multi-camera systems* (Optotrak, Vicon, Qualisys motion capture) exploit advantages of infrared spectrum (infrared emitting diodes) and ensure precision of under 1 mm even at high sampling frequencies [13]. Simultaneous videotape recording and coding ensures intuitive interpretation of infant’s actions. Such combinations have been used for studying reach and grasp development [14], head [15], arm and trunk movement [16, 17]. System disadvantages are high number of needed markers, invasiveness, time-consuming preparation of the measurement subject and measurement system. While system complexity was reduced with marker clusters [18], large segments of missing data due to unexpected infant’s movement and self-occlusion remain a problem [7, 19].

*Electromyography (EMG)* measurements have been used as supplement to optical camera-based experimental setup to extract enhanced movement information and muscle activation data, focusing on studying postural control during infant’s reaching tasks [20, 21].

*Force plates* have been used in combination with movement analysis systems to analyse goal-directed reaching and postural control of healthy infants in supine position [22]. Kyvelidou *et al.* studied sitting postural control, in terms of centre-of-pressure (*COP*) movement, by using force plates alone [23].

*Electromagnetic (EM) tracking systems*[24] have been used in cooperation with motion sensor displacement removal methods [25] to surpass the line-of-sight requirements of optical systems, but suffer from movement limitations due to wiring.

*Accelerometers* have been used to analyse infant’s spontaneous upper [26] and lower extremity movements [27], but do not provide postural information.

*Wireless inertial and magnetic measurement units (IMUs)*[28] are a wearable, non-invasive, low-cost system, consisting of a three-axis gyroscope, three-axis accelerometer, and a three-axis magnetometer. Such set of sensors measures three-dimensional angular velocity, acceleration, and magnetic field vectors. These mechanical sensor signals can be merged using sensor fusion methods to estimate orientation [28–30]. Sensor fusion in this context is covering statistical and deterministic fusion methodology [31] and is needed to overcome the shortcomings of using sensors individually. IMUs have been intensively and reliably used for movement tracking of adults [29, 30, 32], as well as upper extremity motion measurements of primary school children [33]. To the best knowledge of authors, IMU applications for infant movement tracking are rare. Although, Taffoni and colleagues [34] reported of a wired magneto-inertial wearable device design for behavioral analysis of infants, authors presented only preliminary performance results.

*Pressure distribution mattresses* are matrices of usually piezoresistive effect based sensors. Boughorbel *et al.* reported of basic, non-invasive infant trunk posture analysis with feature selection methods. The classification process was performed by majority vote fusion of linear, quadratic, support vector machines (SVM), and k-nearest neighbour (kNN) classifiers [35]. Dusing *et al.* reported of trunk extension and flexion tendencies assessment of infants in supine [36], as well as *COP* movement analysis [37]. More existing applications are in the field of posture analysis of adults, such as non-invasive sleep pattern analysis [38, 39], ulcer prevention methods [40], and posture classification during diagnostic tomography imaging [41]. Despite disadvantages, good reliability and precision are obtained with implementation of data processing and machine learning methods, such as principal component analysis (PCA), SVM [38], kNN [40], Naïve Bayes classifiers, and hidden Markov models [39].

A dedicated multi-sensor based gym for measurement and rehabilitation of pre-term infants is being developed by the FP7 EU project CareToy consortium. As part of the sensor system, a combination of pressure mattresses and IMUs fixed on trunk and arms is proposed for infant’s movement recognition and motor pattern assessment, in view of avoiding the listed drawbacks of other measurement systems.

While the area of sensor based assessment of infant arm motor patterns still lacks a non-invasive, objective, low-cost measurement system, the proposed combination of sensors has not yet been used for such analysis. Therefore, the presented study has several purposes. Firstly, the main intention is to validate the proposed sensor set for analysis of infant arm motor pattern parameters by comparison to referential optoelectronic motion capture (Optotrak) data. The second purpose is to study the dependency of arm kinematic parameters determination in relation to the selected type of IMU sensor placement. Unknown is the influence of system configuration on method precision. Finally, the sensor set is used in a measurement session of a healthy infant to support appropriateness of method and parameters.

## Methods

This section is organized as follows. Initially, the measurement procedure and the experimental setup are presented. Following this, the sensor data processing methodology of trunk and arm posture analysis is described. Finally, the proposed motor pattern parameters are listed, and measurement procedure of the infant is given.

### Experimental setup and measurement procedure

Experimental setup comprised two pressure distribution mattresses, six wireless IMUs, a two-camera optoelectronic measurement system, and a digital video camera.

Two commercially available *pressure distribution mattresses* (CONFORMat System, Model 5330, Tekscan, Inc., USA) were used for pressure distribution measurement. Approximately 80 cm × 47 cm of total pressure sensitive area was covered with 1760 (55 × 32) piezoresistive pressure sensors.

Six *wireless IMUs*, designed exclusively for the EU project CareToy by STMicroelectronics, Italy were used for trunk and arm segment orientation measurements.

Two-camera *optoelectronic measurement system* Optotrak Certus (Northern Digital Inc., Waterloo, ON, Canada) with thirteen infrared emitting diodes as active markers was used for referential measurements of trunk and arm segment positions.

A large number of wired active markers makes validation of the measurement system on an infant practically impossible and ethically controversial. Therefore, a dedicated baby doll, having realistic anthropometric characteristics (trunk weight, arm segment lengths, and elbow joints) of a preterm newborn, was used as a test subject for this purpose.

**Figure 1:**
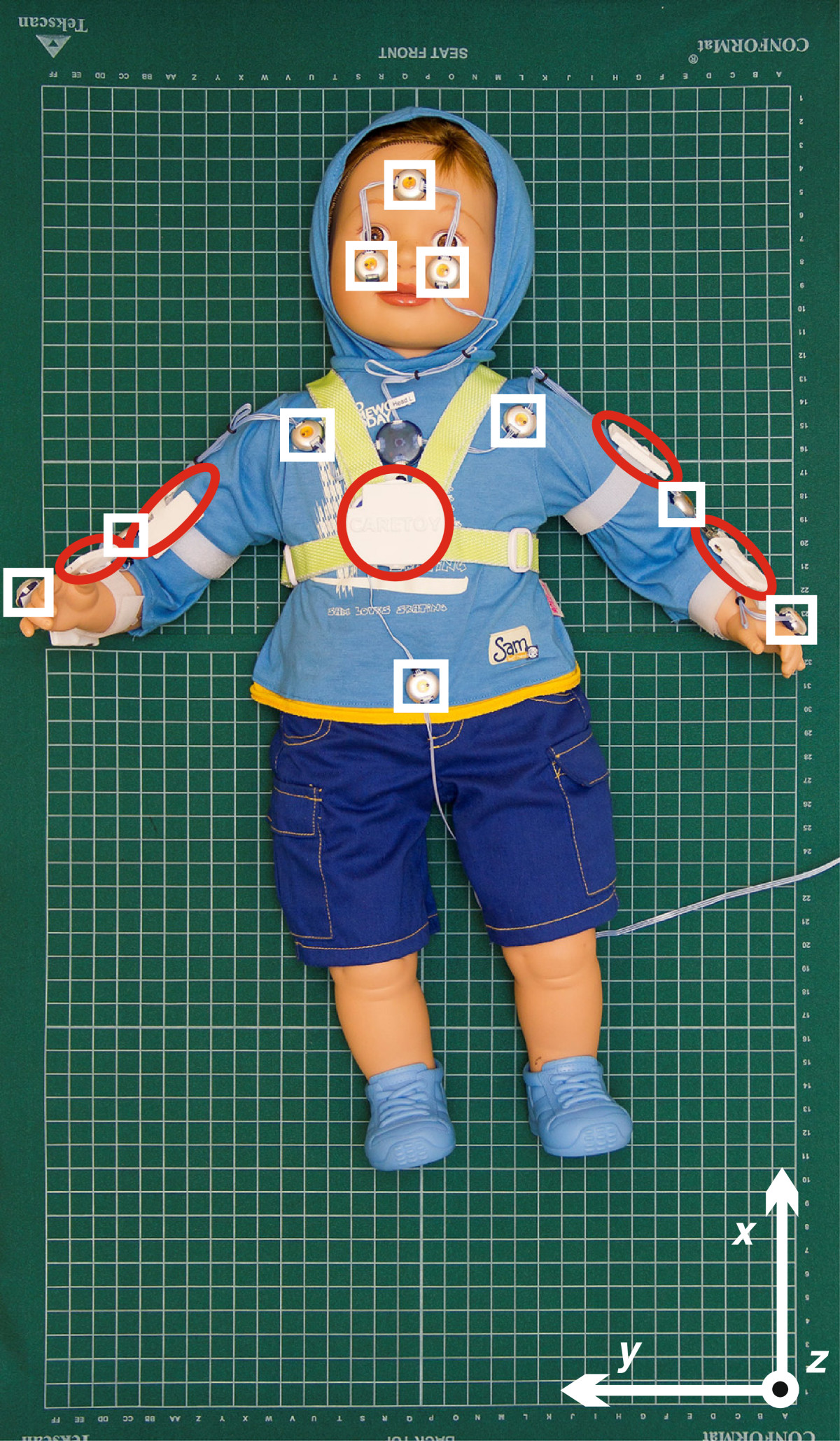
**Experimental setup.** Baby doll is positioned on top of two pressure mattresses, and equipped with five IMU bracelets (red ellipses), positioned one on baby doll’s chest, one on each forearm, and one on each upper arm. Ten Optotrak markers (one on baby doll’s forehead, one on each cheek, one on the frontal side of lower abdomen, one on the frontal side of each shoulder joint, one on the lateral side of each elbow joint, one on the dorsal side of each hand) serve as reference (white rectangles). Referential Optotrak and IMU coordinate system orientation is indicated in the lower right corner (white arrows).

The test subject was equipped with five IMUs (trunk and each arm segment), set inside specially designed silicone bracelets and ten Optotrak markers, fitted on test subject’s anatomical landmarks (Figure 1). One IMU and three Optotrak markers were placed in the corner of the pressure mattress to determine the referential IMU and Optotrak coordinate system (Figure 1). A digital USB video camera was placed above the experimental setup for easier interpretation of numerical results.

MATLAB/Simulink was used for synchronous sensor data acquisition of Optotrak (100 Hz), IMUs (100 Hz), pressure distribution mattress data (30 Hz), and video (10 Hz). Acquired data were stored on a computer hard drive for post processing.

Subject’s arms and trunk were moved by an experienced experimenter similarly to realistic movements of an infant, trying to avoid marker occlusion. Movement activity around the longitudinal (cranial-caudal) axis of the trunk was simulated as rolling over from back to side position in a corkscrew fashion [42]. Simultaneously, spontaneous arm movements were performed on the frontal side of coronal plane around cranial-caudal, ventral-dorsal, and medial-lateral axis. Realistic goal-oriented reach to grasp behaviour was simulated by changing the elbow angle from elbow flexion to elbow extension and vice versa. Average movement speed was approximately 13 cm/s, similar to [43].

### Sensor data processing

**Figure 2:**
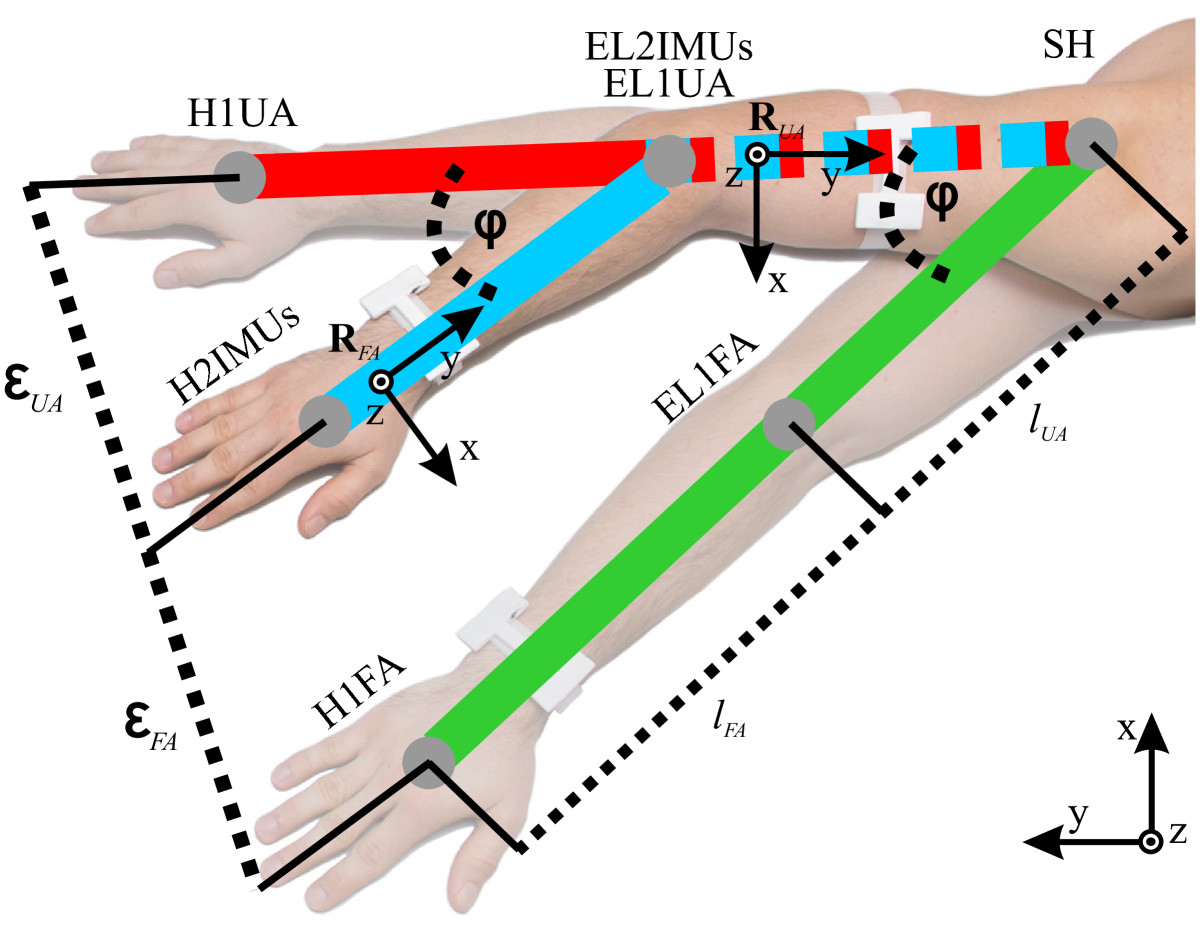
**Arm kinematics.** 2 IMUs per arm (blue lines), only 1 IMU on the upper arm (red lines), and only 1 IMU on the forearm (green lines) sensor placement options are presented. SH, EL, and H represent shoulder, elbow, and hand positions, respectively. *φ* stands for the elbow flexion angle, *l*_*UA*_ and *l*_*FA*_ represent upper and forearm segment lengths, while *ε*_*UA*_ and *ε*_*FA*_ stand for Euclidean distances of upper and forearm sensor placement simplifications. **R**_*UA*_ and **R**_*FA*_ indicate upper and forearm coordinate systems.

Several coordinate systems are used for trunk and arm posture description. *Earth coordinate system (E)* is defined with orientations of gravity and Earth magnetic field vectors. *Trunk coordinate system (T)* is defined with directions of medial-lateral (*x*), caudal-cranial (*y*), and dorsal-ventral (*z*) axes. *Arm segment coordinate systems (upper arm - UA, forearm - FA)* are defined with posterior-anterior (*x*), proximal-distal (*y*), and medial-lateral (*z*) axes (Figure 2). *Referential Optotrak and IMU coordinate system* is defined with orientation of the pressure mattress (Figure 1). In example, $R_{\text{FA}}^{T}$ presents forearm coordinate system orientation, expressed in trunk coordinate system.

#### Trunk posture analysis

Trunk posture analysis comprises data pre-processing and sensor data fusion. Pressure distribution data is a digital grayscale image (55 pixels × 32 pixels) and can be processed with effective digital image processing techniques.

**Figure 3:**
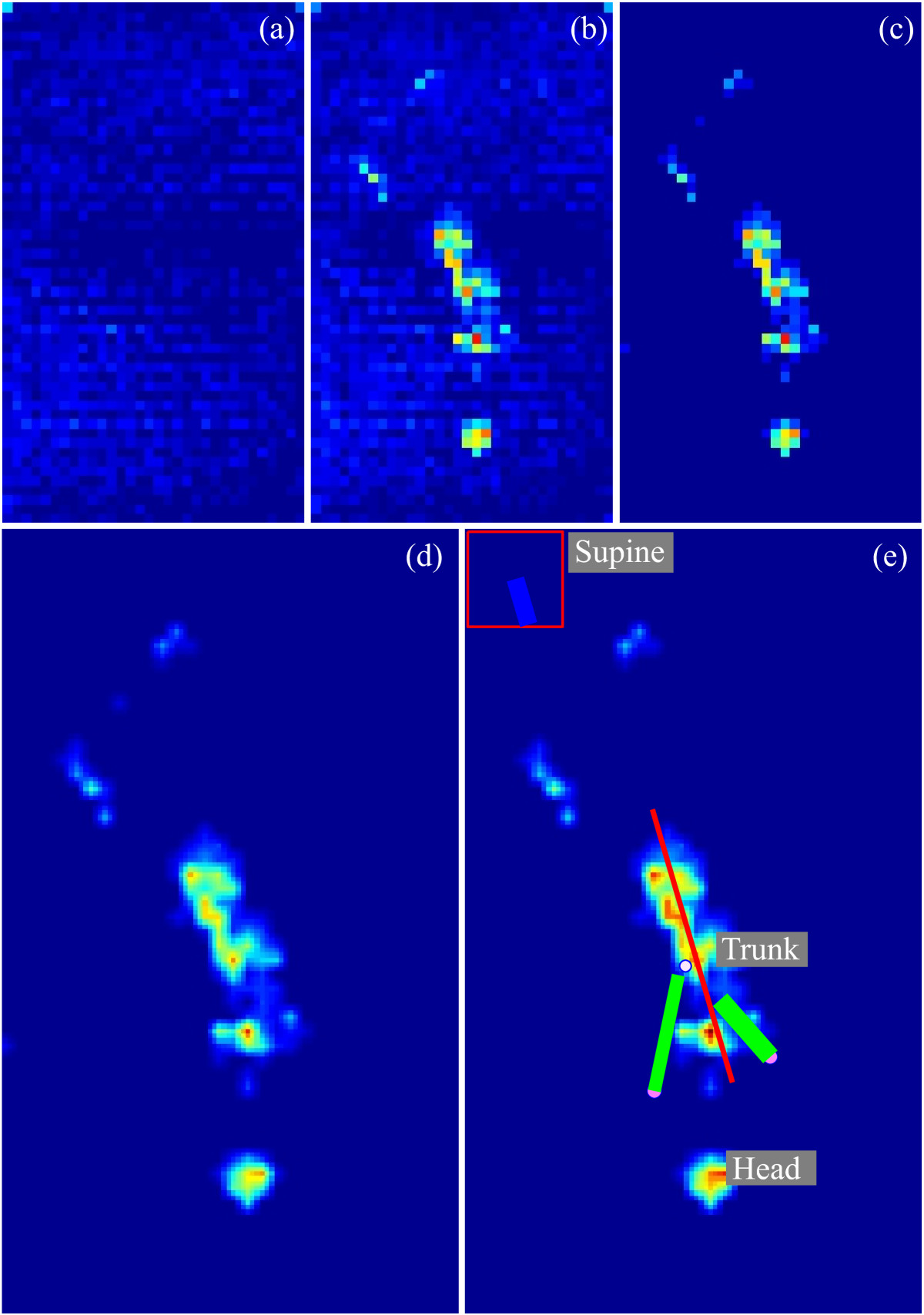
**Pressure data processing.** Bias values matrix **(a)**, loaded matrix before **(b)** and after **(c)** noise removal, and matrix after interpolation **(d)**. **(e)** depicts final data processing results with labelled trunk and head imprints, arm orientation (green lines), trunk orientation (red line), *C* *O* *P*_*mat*_ (white circle), and shoulder positions (purple circles).

Pressure mattress modules have unique default offset level, dependent of the surrounding temperature. Bias values reach up to twenty percent of pixel value range and are noticeable on the loaded pressure distribution matrix (Figure 3b). Therefore, a bias values matrix (Figure 3a), recorded on a regular basis, is used for offset data removal. Noise values, such as oscillations of the output, are removed by data comparison to pre-set thresholds.

A two dimensional eight-connected neighborhood connectivity algorithm of the built-in MATLAB function *bwconncomp* is used to group pressure data into objects. Object properties, such as area, load, and values of minimally and maximally loaded pixels are calculated with the built-in function *regionprops*, and compared to pre-set thresholds for thorough removal of small artefacts (cross-talk).

Successful noise and offset removal ensures extraction of the infant’s pressure imprint matrix (Figure 3c). Linear interpolation method, based on triangles formed by Delaunay triangulation [44], provides higher resolution and accuracy of processed images, resulting in pressure distribution image of 165 pixels × 96 pixels (Figure 3d).

Pressure distribution matrix centre-of-pressure (*COP*_*mat*_) coordinates are calculated with regard to pixel load values and coordinates. Orientation of IMUs, relative to *E* is determined with the Unscented Kalman filter (UKF). UKF is a sensor fusion algorithm for estimation of nonlinear systems and represents an upgrade to the more frequently used extended Kalman filter [28, 29, 45]. *T* orientation is expressed relative to referential IMU coordinate system and is a good estimate of infant’s orientation on the pressure mattress. The trunk IMU can occasionally be displaced and minimally rotated during a measurement session. Therefore, determination of infant’s trunk orientation on the pressure mattress is improved by combined use of trunk IMU and pressure data. Trunk imprint on the pressure mattress is recognized as combination of pixels and objects in close surroundings of *COP*_*mat*_. Two-dimensional trunk imprint orientation on the pressure mattress is extracted with contrast enhancement method [46] and central image moments calculation [47]. Trust levels of acquired data are determined considering trunk imprint load and length, and distance of *COP*_*mat*_ to centre of the pressure mattress. According to the extracted trust level and the determined two-dimensional trunk imprint orientation, trunk IMU orientation is adjusted in order to ensure precise and exact three-dimensional orientation of infant’s trunk on the pressure mattress. Whenever trust levels are too low (insufficient trunk imprint load or length), trunk IMU orientation is adjusted with last reliable offset data.

Approximate three-dimensional shoulder coordinates on the pressure mattress are determined using pre-set distances. Rolling, trunk flexion, and extension activity is detected by the trunk IMU and considered as the shoulder position adjustments. Coordinates are adjusted with respect to the occurring activity in direction towards or away from the trunk midline, as well as towards or away from the trunk centre-of-pressure. Whenever possible, double-histogram analysis, similar to [41], is performed on trunk imprint data to acquire position of shoulders on the pressure mattress. This feature improves precision of the shoulder determination phase and is especially important in case of distinct rolling activity.

#### Arm posture analysis

Pre-multiplication (1) is used to express upper $\left(R_{\text{UA}}^{T}\right)$ and forearm $\left(R_{\text{FA}}^{T}\right)$ segment orientations relative to the adjusted and improved *T*. In (1), **R** is the rotation matrix, while *T*, *E*, and *A* represent trunk, Earth, and arm segment coordinate systems (*UA* and *FA*), respectively.

1 $$R_{A}^{T}=R_{E}^{T}\cdot R_{A}^{E}$$

Elbow (**p**_*EL*_) and hand (**p**_*H*_) position, describing full arm kinematics (Figure 2), can be calculated with (2), using upper $\left(R_{\text{UA}}^{T}\right)$ and forearm $\left(R_{\text{FA}}^{T}\right)$ orientation data (relative to *T*), and arm segment length vectors ($l_{\text{UA}}=\;{\left[\;0,l_{\text{UA}},0\right]}^{T},l_{\text{FA}}=\;{\left[\;0,l_{\text{FA}},0\right]}^{T}$). *l*_*UA*_ and *l*_*FA*_ represent upper and forearm segment lengths.

2 $$\begin{array}{cc} \;p_{\text{EL}}=R_{\text{UA}}^{T} & \cdot l_{\text{UA}}\\ p_{H}=p_{\text{EL}}+R_{\text{FA}}^{T} & \cdot l_{\text{FA}} \end{array}$$

Since a simple system is needed, arm kinematics can be described with only upper or forearm IMU sensor orientation information (Figure 2). Consequently, $R_{\text{UA}}^{T}$ and $R_{\text{FA}}^{T}$ in (2) become $R_{A}^{T}$ in (3), whereas *A* now represents either upper (*UA*) or forearm (*FA*) coordinate system. The choice depends on the currently analysed system simplification.

3 $${\text{p}}_{H}=R_{A}^{T}\cdot \left(l_{\text{UA}}+l_{\text{FA}}\right)$$

The elbow is a hinge-joint with flexion and extension movements, defined with the angle *φ* (Figure 2). Sensor system simplifications with only 1 IMU per arm therefore mostly result in imprecision of elbow angle and can be described with (4), where *ε*_*UA*_ and *ε*_*FA*_ are the errors (Euclidean distances) of upper and forearm IMU sensor placement options (Figure 2). Error dependencies are a cosine theorem variation and are dependent on arm segment length and elbow flexion angle *φ*.

4 $$\begin{array}{l} \varepsilon_{\text{UA}}=l_{\text{FA}}\cdot \sqrt{2-2\cdot \mathrm{cos\varphi}}\\ \varepsilon_{\text{FA}}=l_{\text{UA}}\cdot \sqrt{2-2\cdot \mathrm{cos\varphi}} \end{array}$$

Optotrak marker positions are transformed to the actual anatomical landmark positions (shoulder, abdomen, head) by recalculation of trunk and head plane normal vectors. Elbow and hand marker positions are expressed relative to *T*. Average distances between Optotrak markers, positioned at anatomical landmarks, can be used for arm segment lengths (*l*_*UA*_, *l*_*FA*_) determination. These lengths can also be determined by segment length measurements. Optotrak centre-of-pressure coordinates (*COP*_*opto*_) are calculated as transformed centre of shoulders and lower abdomen marker positions. Comparison to IMU based results is performed by root-mean-square Euclidean distance values (*RMSE*) calculation.

Considering three-dimensional shoulder coordinates and trunk orientation on the pressure mattress, elbow and hand coordinates are expressed relative to the pressure mattress. This is important for identification of infant’s interaction with the gym.

Head imprint is recognized with a series of implemented search algorithms, such as adaptive line-of-sight algorithm, histogram analysis, and the object tracking method (Rihar A, Mihelj M, Kolar J, Pašič J, Munih M: Sensory data fusion of pressure mattress and wireless inertial magnetic measurement units, submitted). First, head imprint is identified through analysis of coordinates, load, and area of objects in proximity of shoulder coordinates. Whenever head and trunk imprints are connected, head cannot be recognized by using the described algorithm and is determined with double histogram analysis, similar to [41]. Head-tracking algorithm is based on limited dynamics of human head movement and ensures higher reliability and robustness. Relevant anatomical landmark coordinates and recognized significant imprint objects can be presented visually (Figure 3e).

#### Motor pattern parameters

To validate the pressure mattress and IMU data in comparison to normative optoelectronic motion capture (Optotrak) data, typical arm motor pattern assessment parameters were calculated. Among these are mean absolute jerk, root-mean-square jerk, spectral arc length [48], root-mean-square acceleration, normalized arm workspace surface envelope area [49], normalized arm workspace volume, reachable workspace volume, travelled path [50], and the hand average speed [51].

*Hand velocityv*_*H*_ is determined with (5), where *w*_*UA*_, *w*_*FA*_, **r**_*UA*_, and **r**_*FA*_ denote the upper and forearm angular velocities, shoulder to elbow (upper arm), and elbow to hand (forearm) vectors, respectively. Angular velocities and acceleration data are measured by the IMU gyroscopes and accelerometers and are expressed relative to the referential coordinate system.

5 $$v_{H}=w_{\text{UA}}\times \left(r_{\text{UA}}+r_{\text{FA}}\right)+\left(w_{\text{FA}}-w_{\text{UA}}\right)\times r_{\text{FA}}$$

In case of only forearm IMU use, *v*_*H*_ is calculated as cross product of forearm angular velocity *w*_*FA*_ and forearm vector **r**_*FA*_. *v*_*H*_ is filtered with a cut-off frequency of 6 Hz [21, 22, 52].

*Dynamic acceleration* of IMU is determined with gravity deduction from the acceleration vector, expressed in the referential coordinate system.

*Jerk* is calculated as the first derivative of acceleration.

Hand velocity *v*_*H*_ can be calculated by integration of IMU dynamic acceleration, but due to acceleration data bias, velocity tends to drift. Such approach is appropriate only for velocity calculation of arm movements with shorter time periods, such as reach to grasp and similar arm movements. Band pass filter eliminates the low frequencies and resolves the drift related problems.

*Spectral arc lengthSAL* metric is appropriate for movement smoothness assessment and was calculated for 150 determined arm movement intervals with (6), where *V*(*w*) is the Fourier magnitude spectrum of *v*_*H*_, and [ 0,*w*_*c*_] is the frequency band, occupied by the given movement [48].

6 $$\begin{array}{ll} \text{SAL} & =-\int_{0}^{w_{c}}\sqrt{{\left(\frac{1}{w_{c}}\right)}^{2}+{\left(\frac{d\hat{V}(w)}{\text{dw}}\right)}^{2}}\text{dw}\;\\ \hat{V}(w) & =\frac{V(w)}{V(0)}\; \end{array}$$

*Velocity*, *acceleration*, and *jerk* are calculated also from referential motion capture (Optotrak) position data as the first, second, and third derivative, respectively. Derivation is subject to noise, therefore data is filtered with a cut-off frequency of 6 Hz [21, 22, 52].

*Pearson correlation coefficientR* is used to determine correlation of referential motion capture system (Optotrak) and IMU based results.

*Root-mean-square Euclidean distancesRMSE*_*arm*_ are calculated to provide comparison for elbow and hand coordinates, which are obtained by referential motion capture system (Optotrak) as well as determined by combined use of pressure mattress and IMU data.

*Arm workspace* is described with normalized workspace surface area [49] and normalized workspace volume values. In case of 2 IMUs per arm sensor placement, transformation of hand coordinates from Cartesian to spherical coordinate system is performed. Radius values vary over time, therefore optimum workspace radius is calculated with the least squares method [53] and transformation back to Cartesian coordinate system is performed. In case of 1 IMU per arm, orientation of both arm segments is considered identical, resulting in constant hand to shoulder distance, therefore described transformations are unnecessary.

*Workspace surface envelope area* is calculated with the alpha shapes method [54] that determines the concave polygon object of hand kinematic data and its surface area value. Normalization to the frontal hemisphere area, which presents the maximum possible arm workspace surface of an infant’s hand, eliminates the influence of segment length measurement errors and makes inter-subject comparison possible.

*Workspace volume* is determined by processing of hand kinematic data with Delaunay triangulation method [44], the “quickhull” algorithm [55] for determination of convex hull object, and subsequent calculation of the corresponding volume. The value is again normalized to the frontal hemisphere volume.

*Reachable workspace* is described by calculation of concave and convex volume. The first one is calculated with the alpha shapes method [54], while the second one is determined with Delaunay triangulation method [44] and the “quickhull” algorithm [55]. As arm coordinates are expressed relative to the pressure mattress and not relative to the trunk, optimum radius determination and coordinate system transformations are not needed.

*Travelled pathP* is calculated with (7), where *n*, *x*, *y*, and *z* represent number of samples in the time-series data and hand coordinates, respectively.

7 $$P=\overset{n}{\underset{i=2}{\sum }}\sqrt{{\left(x_{i}-x_{i-1}\right)}^{2}+{\left(y_{i}-y_{i-1}\right)}^{2}+{\left(z_{i}-z_{i-1}\right)}^{2}}$$

*Average speedS* is calculated as normalized travelled path with respect to the measurement session duration, which makes inter-hand, inter-session, and inter-subject comparison possible.

### Measures from a healthy infant

To support adequacy of method and parameters, parameters of movements in a healthy, five month old infant were acquired using the dedicated multi-sensor based gym with two pressure mattresses, one referential IMU, and one trunk IMU. 1 IMU per forearm sensor placement was chosen to simplify the measurement procedure. The measurements were performed in compliance with the Helsinki Declaration as part of the FP7 EU project CareToy and were overseen by a child therapist. The measurement protocol was approved by the Italian Ministry of Health (DGDFSC 0066613-P-17/09/2013). Proper informed consent was obtained from the parents, who were present throughout the measurement procedure. Referential video recordings were acquired with a digital USB video camera.

## Results

This section provides the validation results. First, dependency of arm kinematics estimation to IMU sensor placement is presented. The velocity, acceleration, and jerk based motor pattern parameter values are given for referential optoelectronic motion capture (Optotrak) and IMU data. Arm workspace results are provided both, visually and numerically. Following this, reachable workspace volume and travelled path parameters are listed. Finally, measures from the infant are presented.

### Arm kinematics estimation

**Figure 4:**
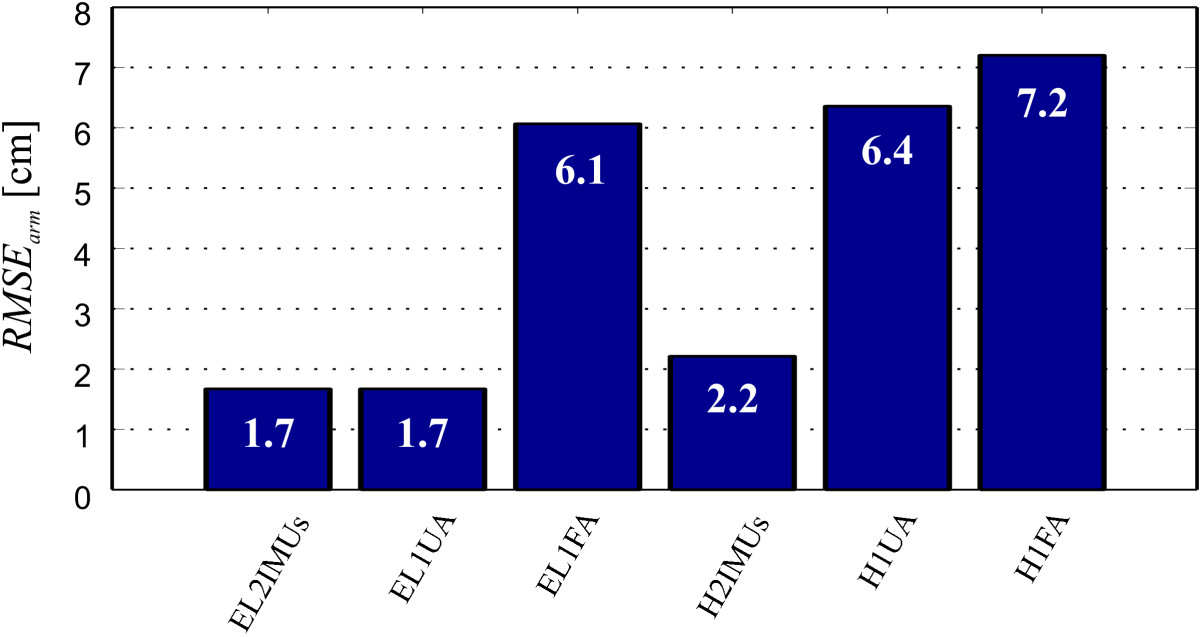
***R*** ***M*** ***S*** ***E***_***arm***_ **values for various IMU sensor placements, compared to referential optoelectronic motion capture (Optotrak) values.** EL2IMUs, H2IMUs, EL1UA, H1UA, EL1FA, and H1FA represent the *R* *M* *S* *E*_*arm*_ values of elbow and hand coordinates for the 2 IMUs per arm, 1 IMU on the upper arm, and 1 IMU on the forearm sensor placements, respectively.

Precision results of arm kinematics estimation are presented in Figure 4. *RMSE*_*arm*_ values of elbow (EL) and hand (H) coordinates are given for all three IMU sensor placement options, compared to referential optoelectronic motion capture (Optotrak) values.

### Motor pattern parameters

Dynamic acceleration and jerk parameters of spontaneous arm movements are shown in Table 1. The values are calculated from referential motion capture (Optotrak) position data and the forearm IMU accelerometer signal. Pearson correlation coefficients *R* are listed for acceleration, jerk, and velocity signals. They are calculated for referential optoelectronic motion capture system (Optotrak) and IMU based approaches.

**Table 1:** **Acceleration, jerk, and velocity based motor pattern parameter results for referential motion capture (Optotrak) and IMU data**

	Optotrak	Forearm IMU
Root mean square acceleration [*m*/*s*^2^]	0.77	0.89
Root mean square jerk [*m*/*s*^3^]	12.08	10.48
Normalized mean absolute jerk [*m*/*s*^3^]	7.98	6.86
Pearson *R* acceleration	0.79	
Pearson *R* jerk	0.76	
Pearson *R* hand velocity forearm IMU	0.93	
Pearson *R* hand velocity 2 IMUs per arm	0.95	

**Figure 5:**
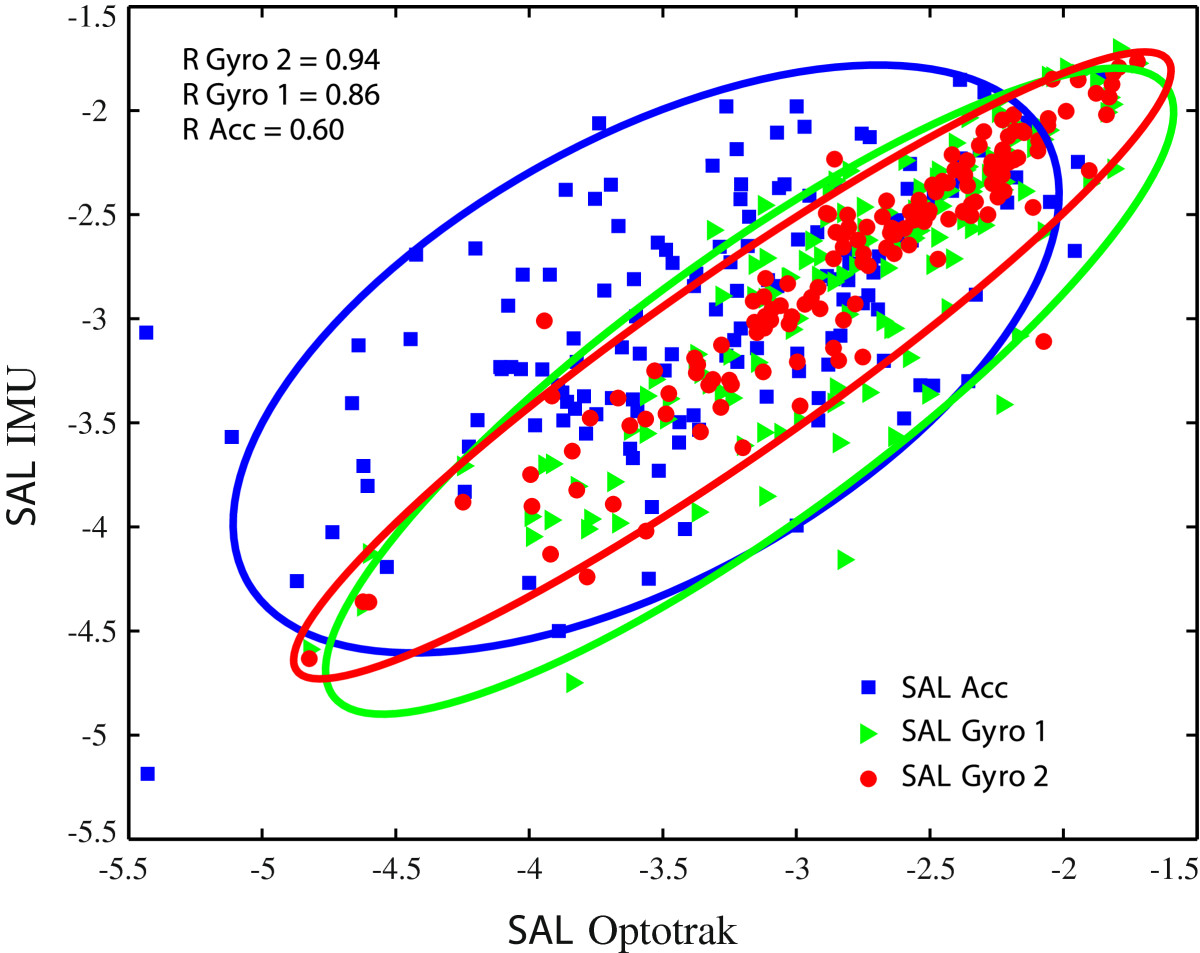
**Spectral arc length (** ***SAL*** **) dependency.** *x* and *y* axes represent *SAL* values, calculated from referential motion capture system (Optotrak) and IMU based hand velocity, respectively. *S* *A* *L* *G* *y* *r* *o* 2 (red circles) and *S* *A* *L* *G* *y* *r* *o* 1 (green triangles) denote *SAL* values of hand velocity, determined from angular velocity for 2 IMUs per arm and 1 IMU per forearm sensor placements, respectively. *S* *A* *L* *A* *c* *c* (blue squares) presents *SAL* results of hand velocity, calculated by integration of forearm IMU acceleration vector. Best fitting ellipses indicate level of linearity. Pearson correlation coefficients *R* for the three possibilities in relation to referential motion capture system (Optotrak) are presented in top left corner.

**Figure 6:**
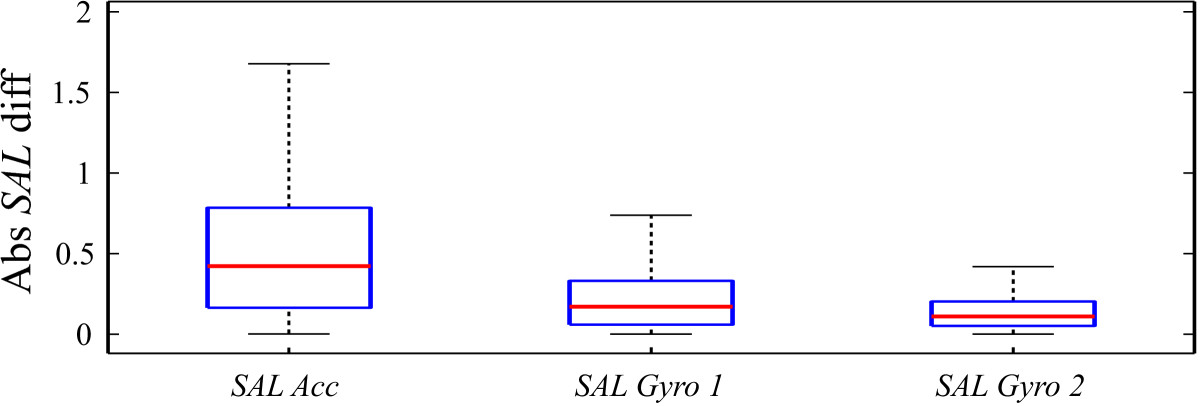
**Absolute differences of** ***SAL*** **results for referential motion capture system (Optotrak) based hand velocity and various IMU based approaches.** First box presents results for hand velocity determination as integral of IMU dynamic acceleration vector (*S* *A* *L* *A* *c* *c*), while second and third box present angular velocity based hand velocity calculation for 1 IMU per forearm (*S* *A* *L* *G* *y* *r* *o* 1) and 2 IMUs per arm (*S* *A* *L* *G* *y* *r* *o* 2) sensor placement.

*SAL* parameter values are presented in Figure 5 and are calculated from referential motion capture system (Optotrak) and IMU based hand velocity of spontaneous arm movements. *SAL* values, based on hand velocity for 2 IMUs per arm sensor placement, are shown along with hand velocity and acceleration integration approach for 1 IMU per forearm sensor placement. Correlation coefficients are given for all three options. Absolute *SAL* differences for referential motion capture system (Optotrak) based hand velocity and the aforementioned IMU based approaches are shown in Figure 6. Box plots are used to present the mean values and level of dispersion.

**Figure 7:**
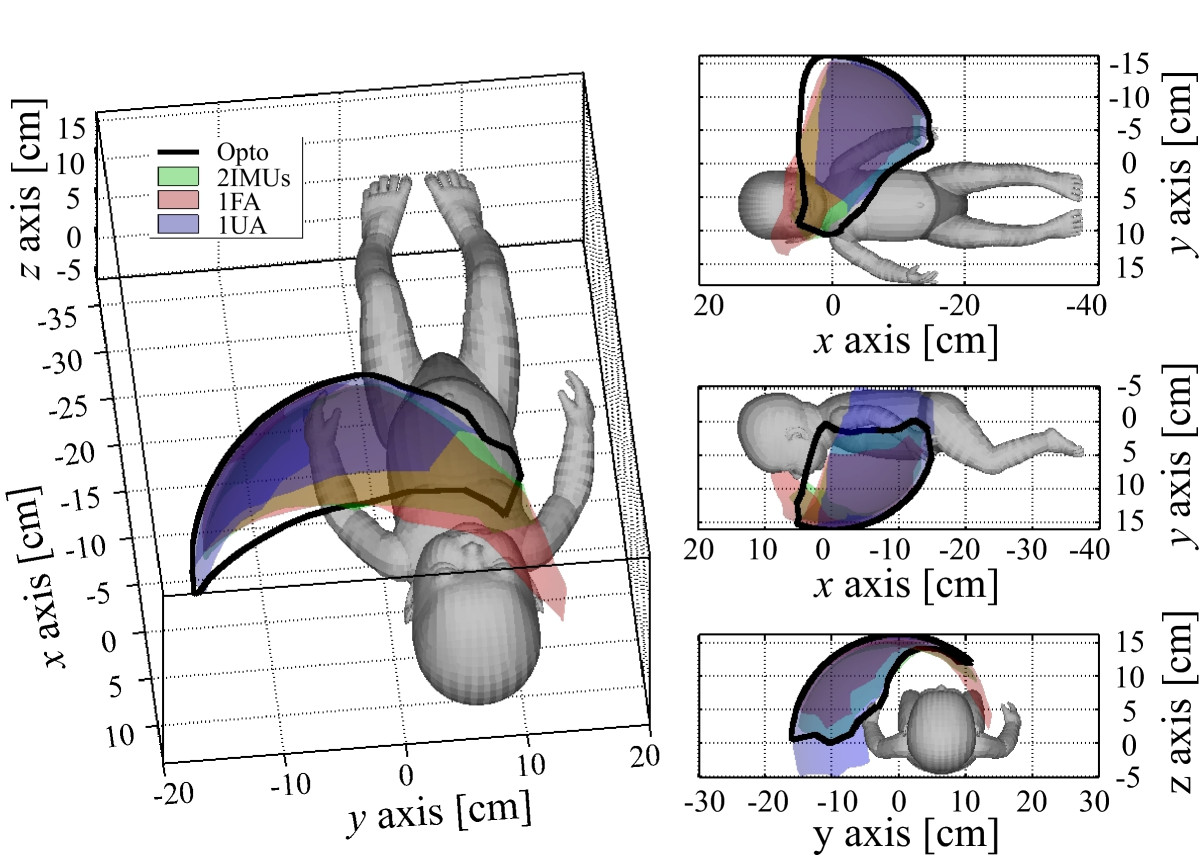
**Left arm workspace surface envelope results.** Results are presented for referential motion capture system (Opto - black line), 2 IMUs per arm (2IMUs - green patch), 1 IMU per forearm (1FA - red patch), and 1 IMU per upper arm (1UA - blue patch) sensor placements. Patches with alternative, mixed colours represent areas, where results overlap. Right half of the figure presents from top to bottom views on baby doll’s coronal, sagittal, and transverse planes.

**Figure 8:**
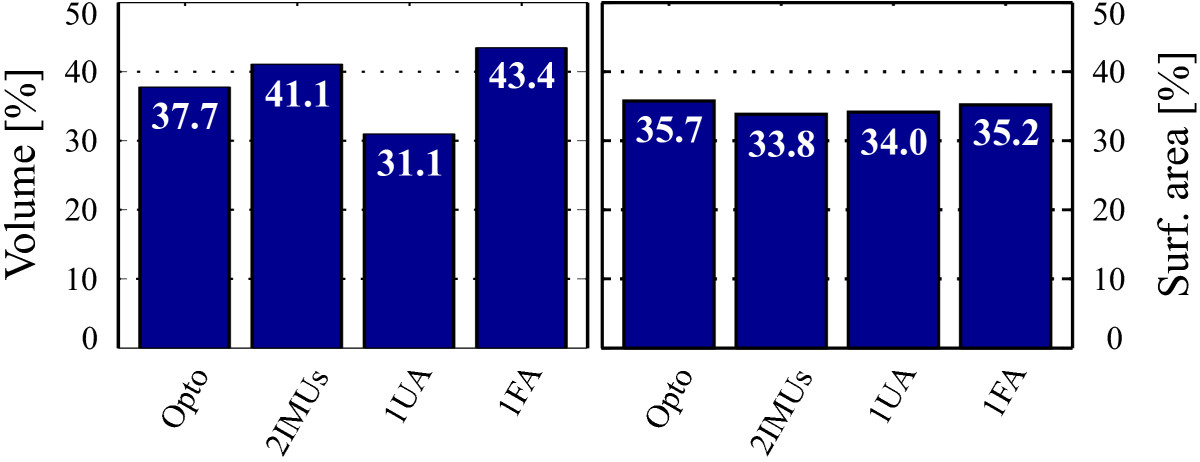
**Normalized workspace volume (left) and normalized surface area (right) values.** Results are presented for referential motion capture system (Opto), 2 IMUs per arm (2IMUs), 1 IMU per upper arm (1UA), and 1 IMU per forearm (1FA) sensor placement.

**Figure 9:**
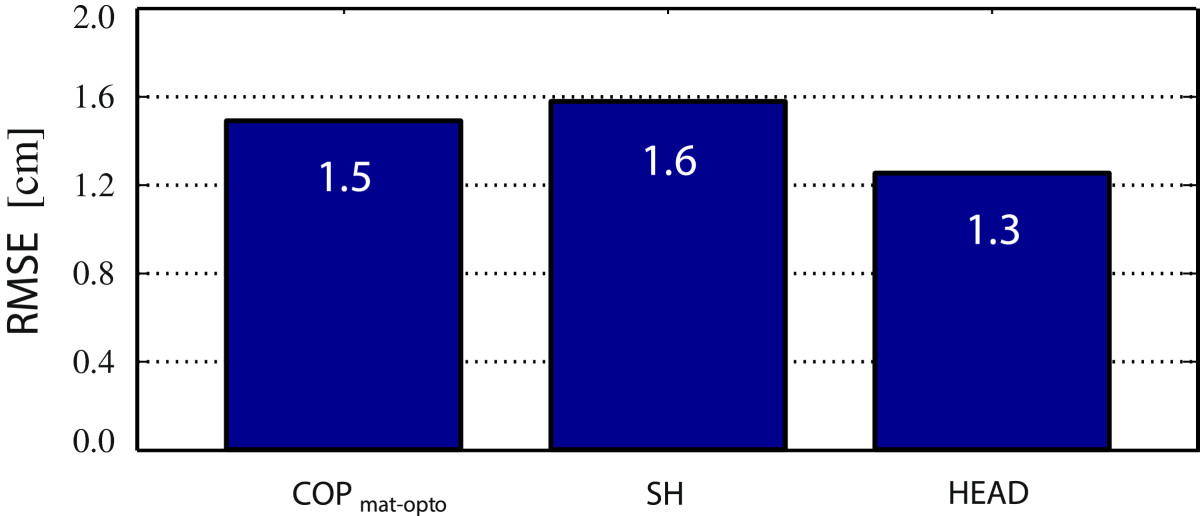
***RMSE*** **values for centre-of-pressure, shoulder and head coordinates.** *C* *O* *P*_*mat*-*opto*_, *SH*, and *HEAD* represent *RMSE* values for centre-of-pressure, shoulder and head coordinates, determined with pressure data processing and with Optotrak.

Arm workspace surface envelope patches for referential motion capture system (Opto), 2 IMUs per arm (2IMUs), 1 IMU per upper (1UA), and 1 IMU per forearm (1FA) sensor placements are presented in Figure 7. Various views on baby doll’s coronal, sagittal, and transverse planes are used to ensure intuitive three-dimensional result interpretation. Figure 8 shows normalized workspace volume and normalized surface area values for the aforementioned approaches. This provides the possibility of volume and surface area percentage comparison. *RMSE* values for centre-of-pressure, shoulders, and head coordinates are given in Figure 9.

Reachable volume space results, along with travelled path *P* and average speed *S* values are presented for the same movements in Tables 2 and 3, respectively. Results are given for referential motion capture system (Optotrak) based approach and three IMU sensor placement options. IMU based results are normalized to referential motion capture (Optotrak) values to ensure intuitive comparison.

**Table 2:** **Reachable volume results for referential motion capture system (Optotrak) and various IMU sensor placements**

Vol. type	Vol. [*c* *m*^3^]	Vol. normalized to Optotrak [%]		
	Opto	2IMUs	1UA	1FA
Concave	2724	96.4	57.7	59.5
Convex	4595	107.4	95.4	131.7

**Table 3:** **Travelled path** ***P*** **and average speed** ***S*** **results for referential motion capture system (Optotrak) and various IMU sensor placements**

	Opto	2IMUs	1UA	1FA
Travelled path *P* [*cm*]	3766	4146	4429	4181
Average speed *S* [ *c* *m*/*s*]	4.5	4.9	5.2	5.0
Average speed norm [%]	100	110	118	111

### Measures from a healthy infant

**Figure 10:**
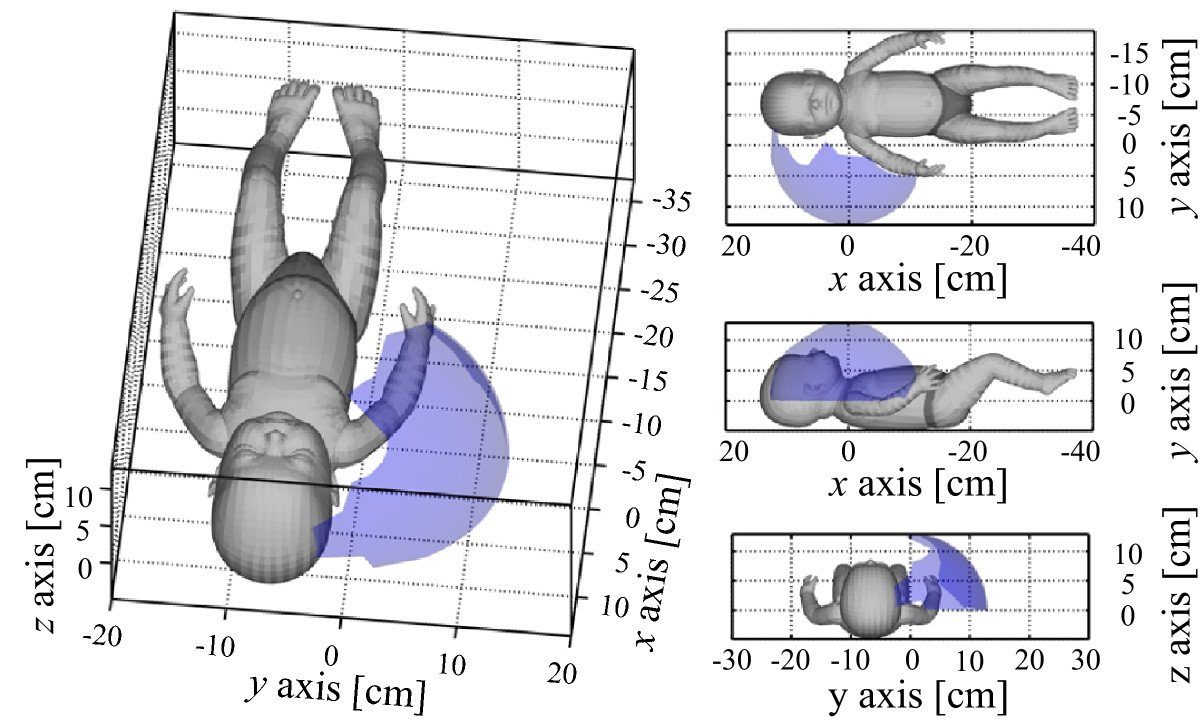
**Right arm workspace surface envelope results for the healthy infant.** Left part of the figure presents the diagonal view, while the right half presents views on the infant’s coronal, sagittal, and transverse planes.

**Figure 11:**
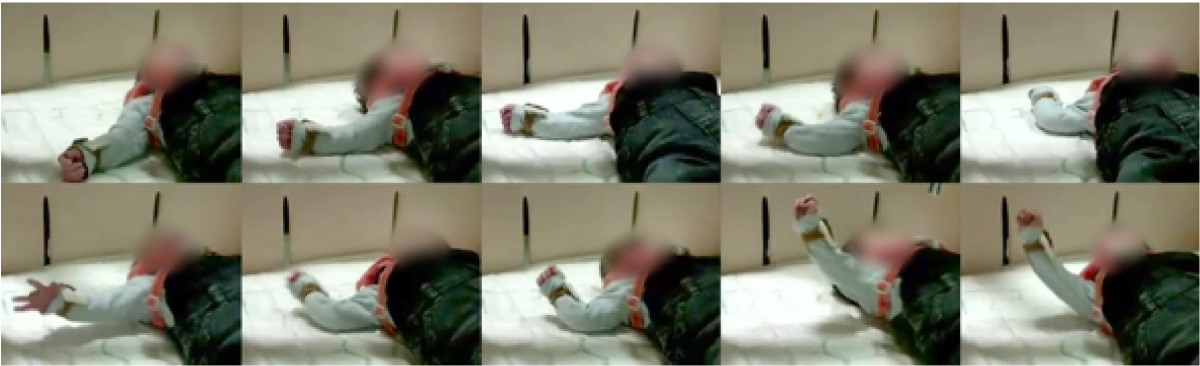
**Referential video recordings.**

Right arm workspace surface envelope results from a healthy infant are presented in Figure 10. Various views are used to ensure intuitive three-dimensional interpretation. Figure 11 in a sequence shows the representative frames of acquired video recordings, which were used for arm workspace validation in Figure 10. Motor pattern parameters of the infant’s movement are given in Table 4. Acceleration and jerk based parameters, *SAL*, arm workspace surface area and volume results are listed along with travelled path *P* and the average speed *S* values.

**Table 4:** **Motor pattern parameters for the measures from a healthy infant**

Root mean square acceleration [ *m*/*s*^2^]	2.9
Root mean square jerk [ *m*/*s*^3^]	39.9
Normalized mean absolute jerk [ *m*/*s*^3^]	20.5
*SAL* mean (standard deviation)	-3.3 (1.0)
Normalized workspace surface area [%]	40.1
Normalized workspace volume [%]	53.0
Travelled path *P* [*cm*]	2397
Average speed *S* [ *c* *m*/*s*]	8.3

## Discussion

This section first presents discussion of arm kinematics estimation values and motor pattern parameter validation results. Following this, a discussion of acquired measures from a healthy infant is given. Finally, advantages of combining sensor data are emphasized.

### Arm kinematics estimation

Elbow (EL2IMUs) and hand (H2IMUs) *RMSE*_*arm*_ values are in range of 2 cm (Figure 4), which confirms that 2 IMUs per arm sensor placement ensures high arm kinematic precision capture. Position errors are a consequence of several factors. Most important are the skin movement and consequently slight displacement of IMU in relation to arm segment orientation and possible incorrect arm segment length measurements. Use of only upper or forearm IMU provides additional measurement system simplification, but directly affects accuracy of elbow and hand position estimation. The reason for this is lack of elbow flexion angle information. In case that only upper arm IMU is used, only hand (H1UA) *RMSE*_*arm*_ values are higher, while in case of forearm IMU use, elbow angle affects accuracy of both anatomical landmarks position estimation (elbow - EL1FA, hand - H1FA) (Figures 2 and 4).

Arm kinematics equations (see Methods) provide description of precision dependency. Euclidean distance values for 1 IMU sensor placements depend on arm segment lengths and elbow flexion angle. Infants under 6 months of age usually have equal upper and forearm segment lengths, therefore in view of extracting precise kinematics this parameter should not affect the choice of sensor placement. Kinematics estimation depends also on the elbow angle, reaching highest precision in case of elbow extension. Infants mostly hold the arms in slight elbow flexion, therefore some level of error is expected in either case of 1 IMU sensor placement. Berthier *et al.*[7] studied that before the reaching onset period, most of the movements are performed with mainly locked elbow angle. The kinematic error should therefore be considerably small. The elbow angle affects kinematic data precision equally for upper and forearm 1 IMU sensor placements, having no effect on the choice of sensor placement. The decision, regarding simplification of system configuration, should be made with respect to other parameter results.

### Motor pattern parameters

Dynamic acceleration and jerk parameter values, calculated from forearm IMU accelerometer deviate from referential motion capture system (Optotrak) based results for less than 20% (Table 1). Referential motion capture system (Optotrak) based acceleration and jerk are determined as second and third derivatives of position. Consequently, the signal noise levels are increasingly high. Despite this, Pearson correlation coefficients for acceleration and jerk are near 0.8. This confirms correlation of both referential motion capture system (Optotrak) and IMU accelerometer based signals. Referential motion capture system (Optotrak) based hand velocity is calculated as the first derivative of position, therefore less noise and thus higher correlation is expected for hand velocity. Correlation coefficients *R* of hand velocity values, determined via angular velocity for 1 IMU per forearm and 2 IMUs per arm sensor placement and by referential motion capture system (Optotrak) are above 0.9 (Table 1). High level of correlation confirms the given hypothesis and verifies adequacy of IMUs for hand velocity studies.

*SAL* results suggest that angular velocity based approach with 2 IMUs per arm (*SALGyro* 2) sensor placement provides best linear dependency results (Figure 5). Pearson correlation coefficient *RGyro* 2 is above 0.9, which confirms the high correlation to referential motion capture system (Optotrak) based *SAL* results (Figure 5). Absolute *SAL* differences are lowest for such approach and have small value dispersion (Figure 6). This verifies appropriateness for movement smoothness assessment. Dependency results for angular velocity based approach with only 1 IMU on the forearm (*SALGyro* 1) are less linear than 2 IMU approach (*SALGyro* 2). Despite this, values still highly correlate to referential motion capture values (*RGyro* 1 above 0.8) with low mean absolute *SAL* difference and modest value dispersion. Lower precision is a consequence of lack of elbow flexion angle information, but such approach is still accurate enough for reliable movement smoothness evaluation. In case of only forearm IMU use and integration of acceleration in order to calculate the hand velocity, *SAL* dependency is least linear of the three options with correlation coefficient *RAcc* of 0.6 (Figure 5). Absolute *SAL* differences are highly dispersed with mean value of almost 0.5 (Figure 6). Higher level of linearity (correlation) is also demonstrated by narrower best fitting ellipses. Results suggest that acceleration based approach is not as suitable for movement smoothness assessment, as angular velocity based approaches.

Arm workspace surface envelope patches for referential motion capture system (Opto), 2 IMUs per arm (2IMUs), 1 IMU per upper (1UA), and 1 IMU per forearm (1FA) sensor placements again confirm that the 2 IMUs per arm sensor placement approach is most similar to referential optoelectronic motion capture data (Figure 7). This is verified also numerically with normalized arm workspace volume and surface area values (Figure 8), which are in general similar for the different approaches. Slight differences presumably arise from incorrect arm segment length measurements and possible IMU displacements. Although, comparison of 1 IMU per arm sensor placement results (Figure 8) confirms elbow angle influence on the surface envelope shape, such approach still offers good insight into arm workspace characteristics (red and blue patches in Figure 7).

*RMSE* values for centre-of-pressure, shoulders, and head coordinates are all under 2 cm, confirming adequacy of incorporated digital image (pressure mattress data) processing techniques for such data extraction (Figure 9).

In case of 2 IMUs per arm sensor placement approach, both concave (96.4%) and convex (107.4%) shape types are appropriate for assessment of reachable volume (Table 2). The values deviate from referential motion capture system (Optotrak) based results for less than 10%. In case that only 1 IMU per arm approach is used, upper arm sensor placement and convex shape determination provide best results (95.4%). Only forearm IMU use is giving less precise reachable volume results (Table 2), which is a consequence of lack of elbow flexion angle data.

As concerned to travelled path *P* and average speed *S* results (Table 3), the 2 IMUs per arm approach is most accurate in comparison to referential optoelectronic motion capture values with estimation error of 10% (normalized average speed value 110%). Results for only 1 IMU per arm sensor placements are less accurate with estimation error under 20% (normalized average speed values 118% and 111%), but still offer insight into infant’s arm and trunk activity. Obtained values represent combined arm and shoulder movement.

Finally, all the evaluation results above confirm that full sensor set, consisting of pressure mattress and 2 IMUs per arm is a reliable substitution to optoelectronic systems for the given application. Motor pattern parameter errors are under 10%, while kinematic estimation error of arm position is less than 2 cm. Along with its simple-to-use character, such system is appropriate for quick, non-invasive, intensive, several times per day measurements of infant kinematics and corresponding motor patterns. Method does not suffer from drawbacks, such as self-occlusion or intolerance to high number of optical markers. Use of simplified system configuration with only 1 IMU per arm does not provide the best possible kinematic precision. Nevertheless, simplicity of use, shorter system preparation time, lower cost, and still acceptable accuracy of motor pattern assessment are convincing for frequent practical use. Approach with only upper arm IMU provides accurate normalized workspace volume and reachable volume results, while normalized workspace surface area, travelled path *P*, and average speed *S* parameters are estimated more accurately with use of only forearm IMU. The latter not only gives better insight into arm’s end-effector (the hand) orientation, but forearm IMU gyroscope and accelerometer additionally ensure good estimation of hand velocity, acceleration, and jerk (Table 1). Accelerometers alone could also be used for hand accelerometry analysis [56]. Therefore, use of only forearm IMU seems more reasonable, sensible, and useful.

### Measures from a healthy infant

Video recordings of arm movement from a healthy infant (Figure 11) were used to perform validation of the determined arm workspace results (Figure 10). Video confirms that the infant held his right arm extended mostly in the lateral, cranial, and ventral-lateral direction. Motor pattern parameter results (Table 4), especially average hand movement speed *S* (8.3 *cm*/*s*), normalized workspace surface area (40.1%), and *SAL* value of -3.3 (1.0) acknowledge that simulated baby doll’s trunk and arm movements were sufficiently similar to movements of real infants. These comparisons demonstrate appropriateness of method and parameters.

### Advantages of combining sensor data

It is important to emphasize that combined use of pressure mattress information and IMU data not only provides higher precision in comparison to using the IMU data alone, but also makes calculation of parameters, describing infant’s activity levels, possible (travelled path *P* and average speed *S*). By incorporating results of infant’s trunk orientation with regard to the pressure mattress, extracted by image moments calculation and digital image processing techniques, performance improvement is accomplished by applying adjustments to the trunk IMU orientation data. Therewith, the determined arm relative to the trunk kinematics is more precise and exact, providing very accurate motor pattern parameter results.

## Conclusions

To conclude, validation results of multi-sensor measurement system comprising two pressure mattresses and IMUs fixed on trunk and arms demonstrate the system usability and precision, while the general approach demonstrates the simplicity to use, high mobility, and non-invasiveness. While use of 2 IMUs per arm provides best results, approach with only 1 IMU per arm is still accurate enough for frequent practical use. Since the system is not limited to laboratory based settings, it could be utilized as part of structured play sessions several times per day at infant’s homes with parents as potential supervisors. Importantly, frequent measurements also reduce the influence of infant’s day-to-day mood and give full insight into infant movement and motor skills. Due to excellent complementarity of system components, the system holds an enviable potential for accurate, sensor based infant trunk posture and arm movement assessment.

## Consent

Written informed consent was obtained from the infant’s parent for the publication of this report and any accompanying images.

## Authors’ original submitted files for images

Below are the links to the authors’ original submitted files for images. Authors’ original file for figure 1 Authors’ original file for figure 2 Authors’ original file for figure 3 Authors’ original file for figure 4 Authors’ original file for figure 5 Authors’ original file for figure 6 Authors’ original file for figure 7 Authors’ original file for figure 8 Authors’ original file for figure 9 Authors’ original file for figure 10 Authors’ original file for figure 11
