# Supplementary figures and images for: Infant trunk posture and arm movement assessment using pressure mattress, inertial and magnetic measurement units (IMUs)
Source: J Neuroeng Rehabil. 2014 Sep 6;11:133. doi: 10.1186/1743-0003-11-133 (PMC4247204; doi:10.1186/1743-0003-11-133)

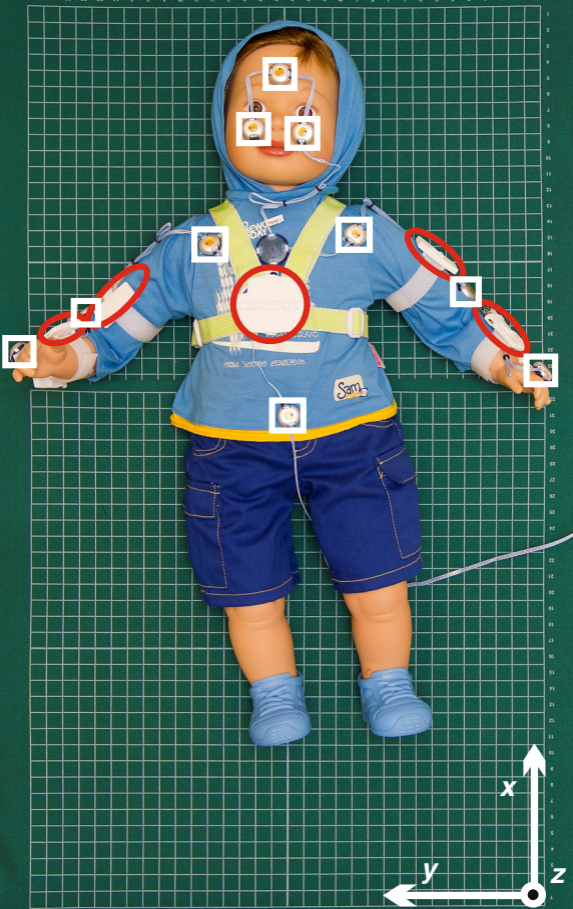

Supplement: Supplementary file 1 — Authors’ original file for figure 1 [file 12984_2014_672_MOESM1_ESM.pdf]

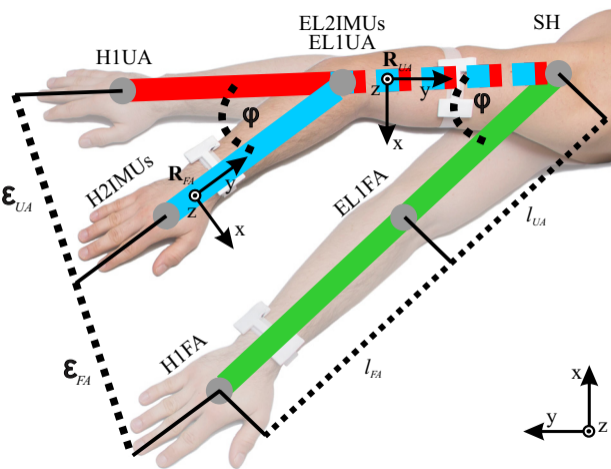

Supplement: Supplementary file 2 — Authors’ original file for figure 2 [file 12984_2014_672_MOESM2_ESM.pdf]

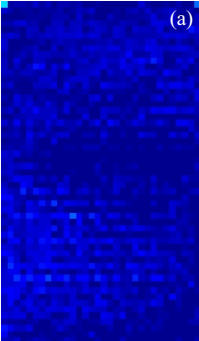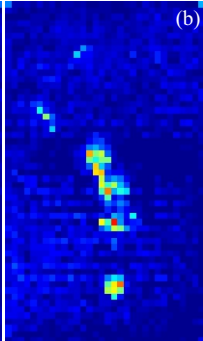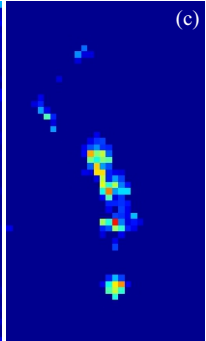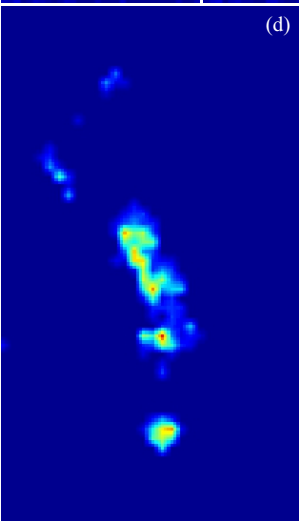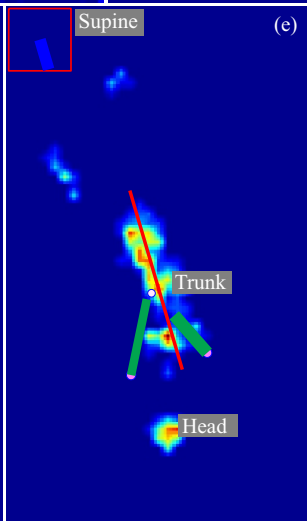

Supplement: Supplementary file 3 — Authors’ original file for figure 3 [file 12984_2014_672_MOESM3_ESM.pdf]

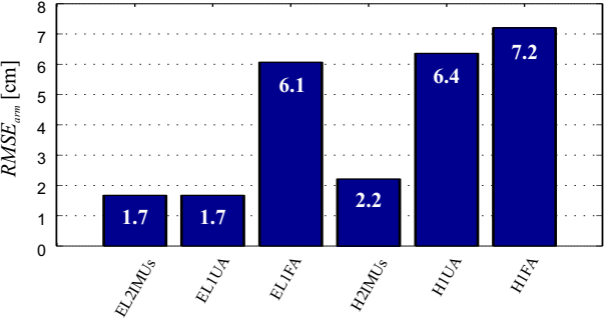

Supplement: Supplementary file 4 — Authors’ original file for figure 4 [file 12984_2014_672_MOESM4_ESM.pdf]

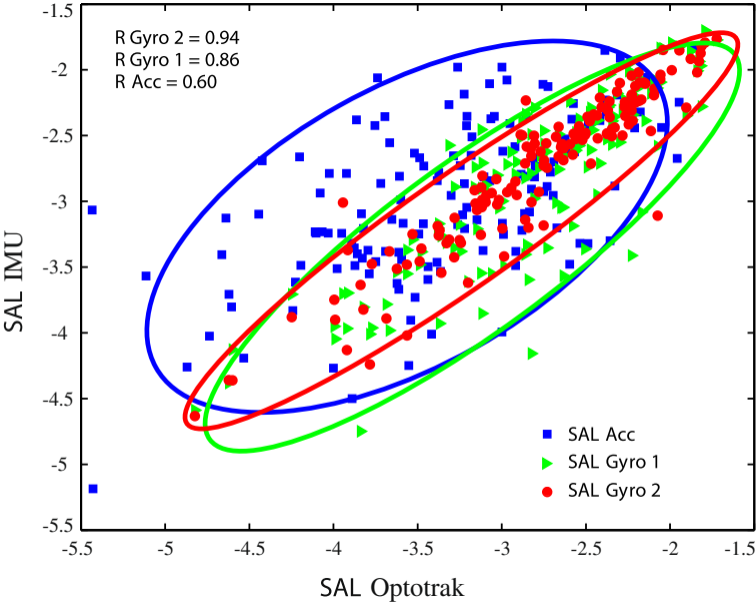

Supplement: Supplementary file 5 — Authors’ original file for figure 5 [file 12984_2014_672_MOESM5_ESM.pdf]

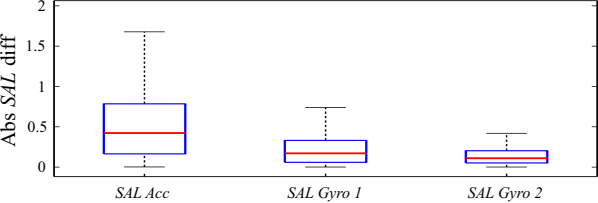

Supplement: Supplementary file 6 — Authors’ original file for figure 6 [file 12984_2014_672_MOESM6_ESM.pdf]

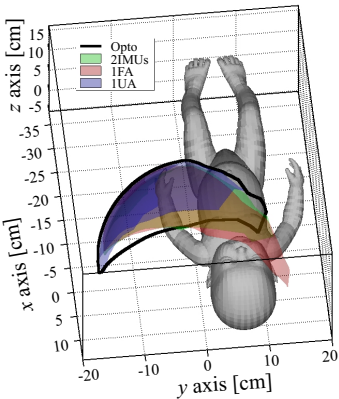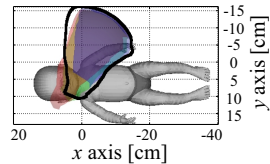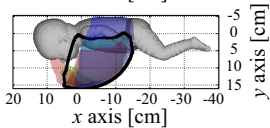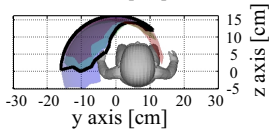

Supplement: Supplementary file 7 — Authors’ original file for figure 7 [file 12984_2014_672_MOESM7_ESM.pdf]

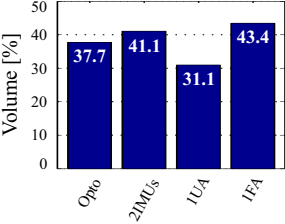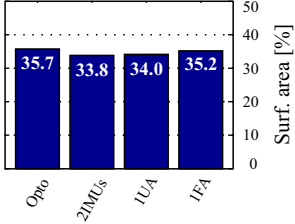

Supplement: Supplementary file 8 — Authors’ original file for figure 8 [file 12984_2014_672_MOESM8_ESM.pdf]

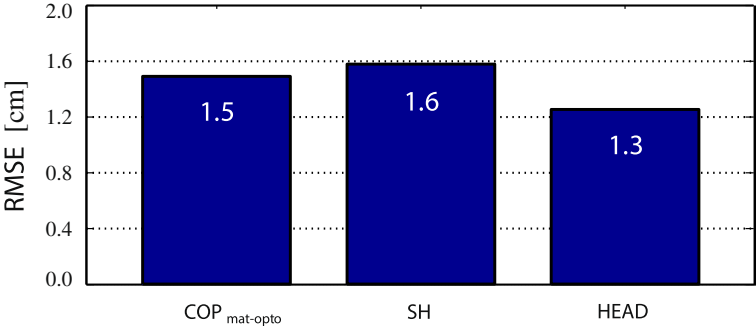

Supplement: Supplementary file 9 — Authors’ original file for figure 9 [file 12984_2014_672_MOESM9_ESM.pdf]

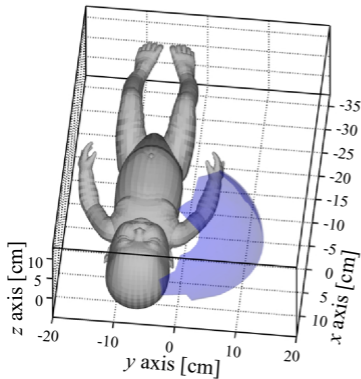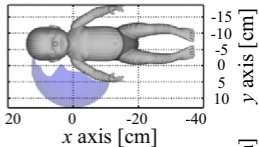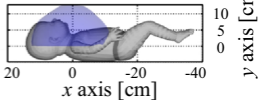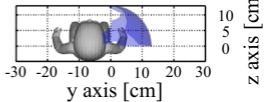

Supplement: Supplementary file 10 — Authors’ original file for figure 10 [file 12984_2014_672_MOESM10_ESM.pdf]

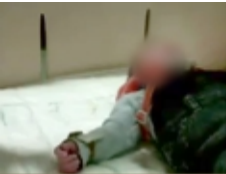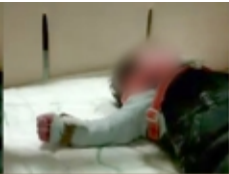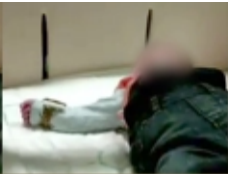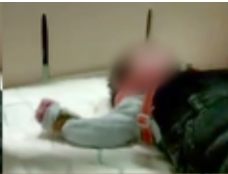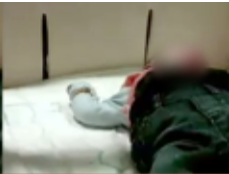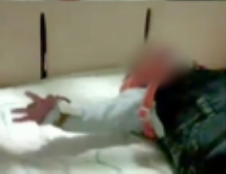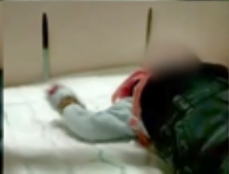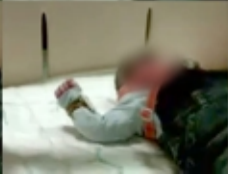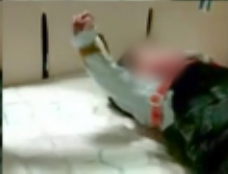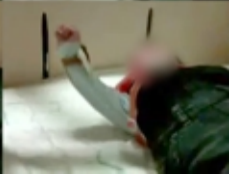

Supplement: Supplementary file 11 — Authors’ original file for figure 11 [file 12984_2014_672_MOESM11_ESM.pdf]
